# Spheroid‐based matrix‐induced autologous chondrocyte implantation combined with autologous bone grafting restores physiological cartilage and subchondral bone properties in osteochondral knee defects—An MRI‐based analysis

**DOI:** 10.1002/ksa.70091

**Published:** 2025-10-15

**Authors:** Stephan Oehme, Benjamin Bartek, Janne Callsen, Jessica Schallock, Tobias Winkler, Thorsten Diekhoff, Tobias Jung, Danko Dan Milinkovic

**Affiliations:** ^1^ Center for Musculoskeletal Surgery, Charité ‐ Universitätsmedizin Berlin Corporate Member of Freie Universität Berlin, Humboldt‐Universität zu Berlin Berlin Germany; ^2^ Berlin Institute of Health at Charité, Julius Wolff Institute Universitätsmedizin Berlin Berlin Germany; ^3^ Berlin Institute of Health Center for Regenerative Therapies, Berlin Institute of Health at Charité Universitätsmedizin Berlin Berlin Germany; ^4^ Department of Radiology, Charité ‐ Universitätsmedizin Berlin, Corporate Member of Freie Universität Berlin Humboldt‐Universität zu Berlin Berlin Germany

**Keywords:** autologous chondrocyte implantation, cartilage, knee, MOCART, osteochondral defects

## Abstract

**Purpose:**

This study aimed to evaluate the radiological and patient‐reported outcome measures (PROMs) following spheroid‐based matrix‐induced autologous chondrocyte implantation combined with autologous bone grafting (MABCI) for the treatment of osteochondral defects of the knee.

**Methods:**

In this retrospective cohort analysis of prospectively collected data, 28 consecutive patients with 29 defects of the femoral condyle (male: 15, female: 13, mean age 27.3 ± 9.8 years, mean defect size 3.7 ± 1.6 cm²) treated with MABCI were included. Radiological assessments were performed using 3T magnetic resonance imaging (MRI) to evaluate cartilage thickness, T2 relaxation times and Magnetic Resonance Observation of Cartilage Repair Tissue (MOCART) 2.0 scores. Clinical outcomes were assessed using validated PROMs, including the International Knee Documentation Committee (IKDC) score, the Knee Injury and Osteoarthritis Outcome Score (KOOS), the Lysholm scale and the Patient‐Reported Outcomes Measurement Information System 29‐Item Profile (PROMIS‐29). Correlation analyses were conducted to evaluate associations between radiological parameters and PROMs.

**Results:**

The analysis was conducted at a mean follow‐up of 44.4 ± 21.6 months. Cartilage thickness in the repair tissue exceeded that of the reference cartilage (3.4 ± 0.9 mm vs. 3.0 ± 0.5 mm; *p* = 0.036), while T2 relaxation times in the repair tissue were within physiological range (50.5 ± 40.0 ms vs. 41.3 ± 5.4 ms; n.s.). The mean MOCART 2.0 score was 61.3 ± 13.0 (volume fill: 16.1 ± 3.7, integration with adjacent cartilage: 12.9 ± 3.3), reflecting satisfactory repair quality. All clinical scores showed significant improvement compared to the preoperative condition (IKDC: 70.6 ± 13.2 vs. 54.8 ± 17.5, *p* < 0.001; KOOS: pain 78.7 ± 16.9 vs. 66.1 ± 18.6, *p* = 0.002, symptoms 75.6 ±  16.0 vs. 65.8 ± 19.1, *p* = 0.006, ADL 86.8 ± 14.0 vs. 77.6 ± 16.8, *p* = 0.003, sport 63.0 ± 24.2 vs. 47.3 ± 26.9, *p* = 0.009, quality of life (QoL) 51.0 ± 22.7 vs. 39.6 ± 17.5, *p* = 0.014; numeric pain rating scale: 3.0 ± 2.2 vs. 5.2 ± 2.4, *p* < 0.001). A greater cartilage thickness was associated with reduced pain intensity (*r* = −0.457, *p* = 0.013) and improved QoL (*r* = 0.429, *p* = 0.020), indicating that better structural repair is associated with improved patient satisfaction.

**Conclusions:**

MABCI results in satisfactory cartilage regeneration and subchondral remodelling in addition to significant improvements in patient satisfaction and overall QoL.

**Level of Evidence:**

Level III.

**Abbreviations**
ABCI: spheroid‐based matrix‐induced autologous chondrocyte implantation
ACI: autologous chondrocyte implantation
CID: clinical important difference
ICC: intraclass correlation coefficient
ICRS: International Cartilage Repair Society
IKDC: International Knee Documentation Committee
IRR: interrater reliability
KOOS: Knee Injury and Osteoarthritis Outcome Score
MABCI: modern, spheroid‐based matrix‐induced ACI
MABCI: spheroid‐based matrix‐induced autologous chondrocyte implantation
MACI: matrix‐induced autologous chondrocyte implantation
MOCART 2.0: Magnetic Resonance Observation of Cartilage Repair Tissue (MOCART) 2.0 scores
MRI: magnetic resonance imaging
OATS: osteochondral autograft transplantation system
PASS: patient acceptable symptomatic state
PROMIS‐29: Patient‐Reported Outcomes Measurement Information System 29‐Item Profile
PROMs: patient‐reported outcome measure scores
QoL: quality of life
SD: standard deviation

## INTRODUCTION

Osteochondral knee defects are a clinical challenge with limited intrinsic healing potential and risk of early osteoarthritis [1, 8, 14, 18]. Successful treatment requires the restoration of both the articular cartilage and the underlying subchondral bone, as these structures function as an integrated unit [1, 8, 18, 30, 34, 38].

Traditional treatment options, such as microfracture and osteochondral autograft transplantation system (OATS), have shown variable long‐term outcomes. Microfracture is minimally invasive but mainly yields fibrocartilage that deteriorates over time [5, 8, 16, 21, 24, 33]. OATS can restore hyaline cartilage but is limited by donor site morbidity and graft availability [4, 8, 17, 18, 25].

Autologous chondrocyte implantation (ACI) is an established option for full‐thickness cartilage defects. The development into matrix‐induced ACI (MACI) improved cell distribution, lowered the risk of hypertrophy and led to better outcomes [11, 25, 30]. To address osteochondral defects ACI combined with autologous bone grafting (ABCI) has been introduced [11, 25, 30]. Systematic reviews have demonstrated favourable mid‐ to long‐term clinical outcomes following ABCI [1, 18, 19, 24, 26, 27, 30].

Despite the growing evidence supporting ABCI, data on matrix‐induced ACI techniques in combination with autologous bone grafting (MABCI), remain limited, even though their use in clinical practice is increasing. Advanced MR sequences, such as T2 mapping or sophisticated scores, for example, the Magnetic Resonance Observation of Cartilage Repair Tissue 2.0 (MOCART 2.0), offer valuable insights into the quality of the regenerated tissue and the biological processes underlying osteochondral repair [4, 12, 15, 16, 35].

However, only a limited number of studies have applied these techniques to systematically assess both radiological and clinical outcomes following MABCI for osteochondral defects of the knee, and none have specifically examined the spheroid‐based MABCI technique. Consequently, the primary aim of this study was to comprehensively analyse the radiological and patient‐reported outcomes following spheroid‐based MABCI for the treatment of osteochondral defects of the knee. Additionally, it was sought to explore the correlation between various morphological characteristics of the repair tissue and subchondral bone with patient‐reported outcome measures (PROMs). It was hypothesised that MABCI would restore cartilage and subchondral bone properties towards physiological values and that physiological radiological outcomes would correlate with improved PROMs.

## METHODS

A retrospective analysis of prospectively collected data of patients who underwent MABCI for osteochondral defects of the femoral condyle at a single university centre between September 2014 and August 2020 was performed. Inclusion criteria were symptomatic osteochondral defects of the knee classified as International Cartilage Repair Society (ICRS) grade 4, confirmed via MRI and arthroscopic evaluation at index surgery. Patients were required to be aged ≥18 years at the time of follow‐up with a minimum follow‐up of 1 year postoperatively. Exclusion criteria were defined to minimise potential confounding factors that could impair osteochondral healing and bias the interpretation of clinical and radiological outcomes. Namely, patients with significant meniscal injuries affecting more than one‐third of the meniscal tissue or with ligamentous insufficiency were excluded because these conditions may compromise the healing process of the osteochondral reconstruction. Further, patients with neoplastic diseases or pregnancy were excluded as these systemic conditions may influence both radiological findings and functional outcomes as well.

A total of 28 patients with 29 osteochondral defects of the femoral condyle who underwent spheroid based MABCI were included in this study. Six patients were excluded based on the defined exclusion criteria. One had a preoperative diagnosed neoplastic disease, two had a meniscal injury involving more than one‐third of the meniscus, two patients were pregnant, and one patient had a conversion to a patient specific small metal implant, which was considered a clinical failure. Detailed information is provided in the CONSORT diagram (Figure 1). The mean age at the time of surgery was 27.3 ± 9.8 years, ranging from 15 to 50 years, with a nearly equal gender distribution (46.4% female and 53.6% male). The majority of defects were located on the medial femoral condyle (79.3%), while the lateral femoral condyle accounted for 20.7% of cases. The mean defect size was 3.7 ± 1.6 cm², with 41.4% of defects smaller than 3.5 cm² and 58.6% larger. Prior surgeries were performed in seven patients (25%). Concomitant procedures were performed in three patients (10.7%), including high tibial osteotomy (*n* = 2) and MACI in the patellofemoral joint (*n* = 1). The detailed characteristics of the study population are summarised in Table 1.

**Figure 1:**
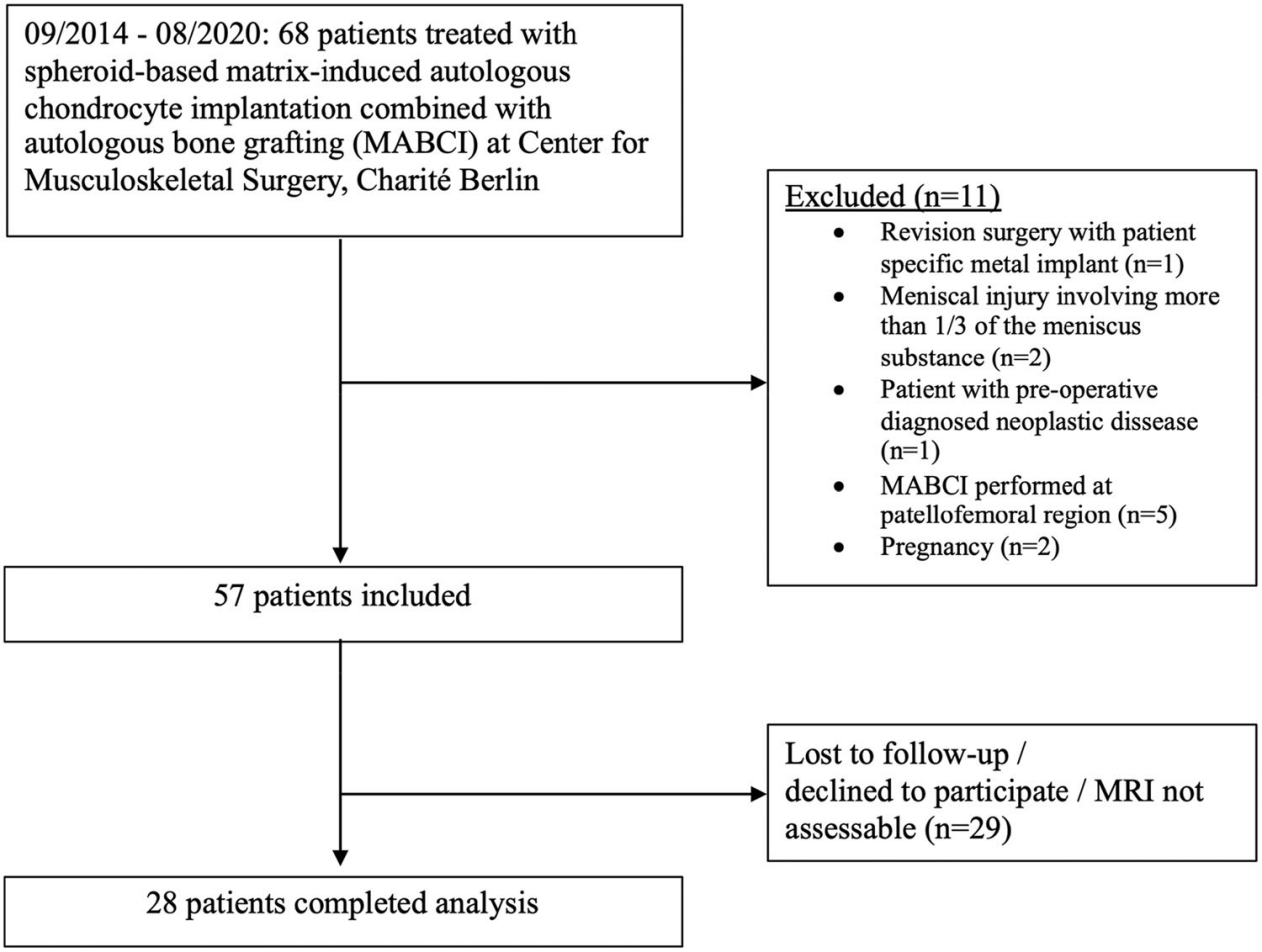
CONSORT diagram of patient acquisition.

**Table 1:** Demographic data of the patient cohort.

Variable	Value
Number of patients	28
Number of knees treated	29
Mean age at surgery (years)	27.3 ± 9.8
Age range	15–50
Age ≤ 20 years	*n* = 10 (35.7%)
Age 21–30 years	*n* = 8 (28.6%)
Age ≥ 31 years	*n* = 10 (35.7%)
Sex (female)	*n* = 13 (46.4%)
Sex (male)	*n* = 15 (53.6%)
Defect location: Medial femoral condyle	*n* = 23 (79.3%)
Defect location: Lateral femoral condyle	*n* = 6 (20.7%)
Mean defect size (cm²)	3.7 ± 1.6
Defect size range (cm²)	1.1–7.0
Defect < 3.5 cm²	*n* = 12 (41.4%)
Defect ≥ 3.5 cm²	*n* = 17 (58.6%)
Mean follow‐up time (months)	44.4 ± 21.6
Follow‐up < 5 years	*n* = 17 (60.7%)
Follow‐up ≥ 5 years	*n* = 11 (39.3%)
Prior surgeries	*n* = 7 (25%)
	2x OAT; 2x MFX, 1x ABG, 1x allogenic BG, 1x FBR
Concomitant surgeries	*n* = 3 (10.7%)
	2x HTO, 1x ACI PF

Abbreviations: ABG, autologous bone graft; ACI, autologous chondrocyte implantation; HTO, high tibial osteotomy; MFX, microfracture; OAT, osteochondral autograft transplantation; PF, patellofemoral.

### Surgical technique

MABCI was performed as a two‐step procedure. The initial stage involved diagnostic arthroscopy to confirm the extent of the osteochondral defect and to obtain cartilage biopsies from nonweight‐bearing cartilage zone in the intercondylar femoral notch. These biopsies were sent to the laboratory (CO.DON® AG) for chondrocyte expansion and spheroid formation [3]. Approximately 35–55 days after the first surgery, bone grafting in combination with matrix‐induced spheroid‐based chondrocyte implantation was performed under general anaesthesia with the use of a tourniquet. A mini‐open arthrotomy was performed and the osteochondral defect was debrided, removing any sclerotic subchondral bone with ring curettes. The defect bed was prepared to a stable, vascularises base. Autologous bone plugs were harvested from the proximal tibia or distal femur using an osteochondral autograft transfersystem (Arthrex Co, Naples) and impacted into the defect site to restore subchondral integrity. One to five cortico‐cancellous bone plugs, each measuring 10 mm in diameter, were harvested depending on the lesion size, and the donor site was subsequently filled with allogenic cancellous bone. Subsequently, the cultured spheroid‐based chondrocyte aggregates were implanted onto the grafted area, adhering without the need for membranes or additional fixation [31, 39] (Figure 2). If concomitant procedures were required, such as high tibial osteotomies, they were performed during the second surgery.

**Figure 2:**
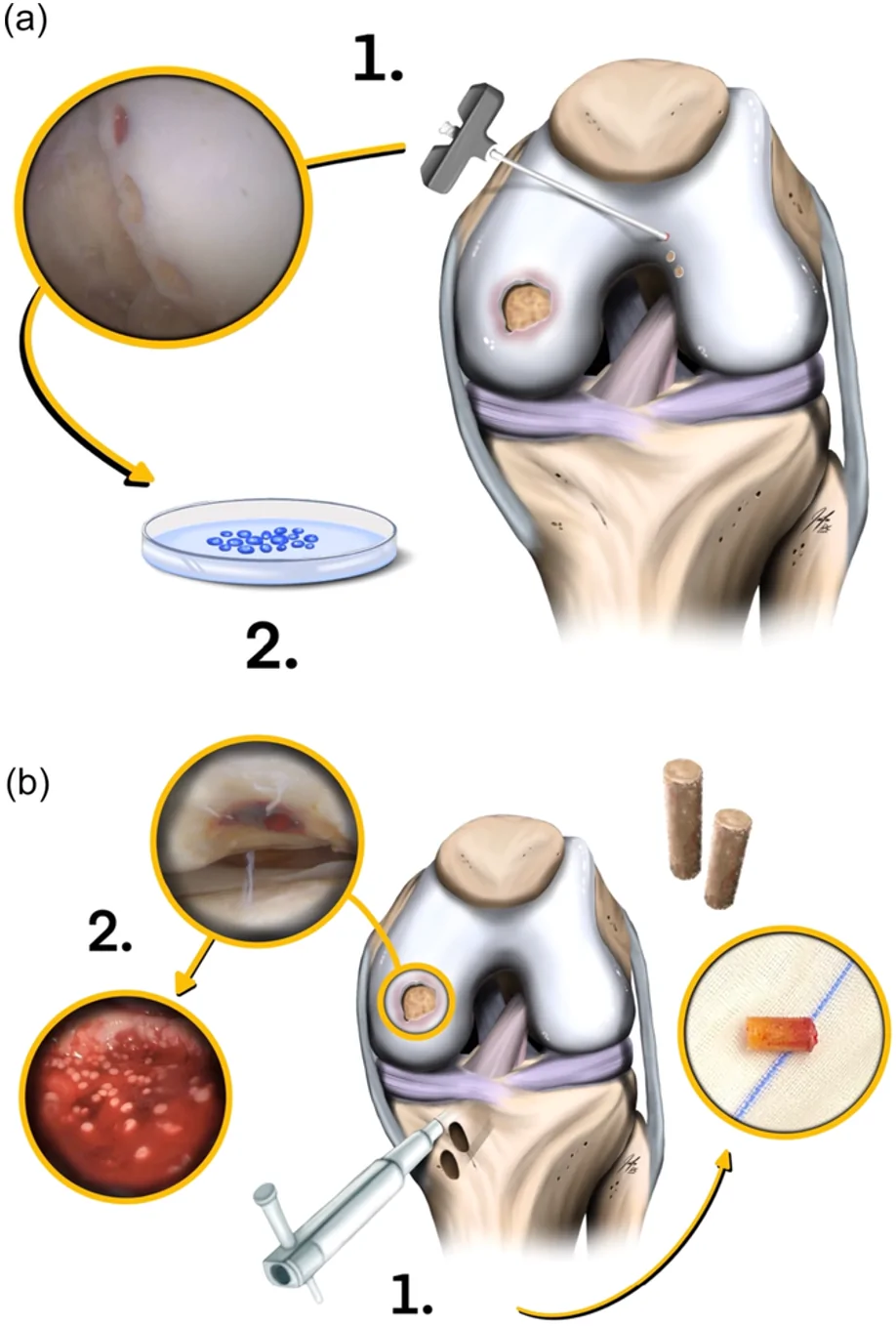
Surgical procedure. (a) 1: cartilage biopsy, 2: chondrocytes in vitro cultivation; (b) 1: autologous bone harvesting, 2: autologous bone grafting in the defect, followed by spheroid‐based matrix‐induced autologous chondrocyte implantation.

Postoperatively, a standardised rehabilitation protocol was followed. After the first surgery, partial weight‐bearing was maintained for 1 week without any restrictions to the range of motion. After the second surgery, partial weight‐bearing was continued for 6 weeks, with active and passive knee flexion limited to 60° during the first 2 weeks, followed by a limitation of active flexion to 90° until the end of the 6th week. All patients underwent intensive physical therapy after the second surgery for the duration of up to 3 months postoperatively.

### Radiological assessment

Radiological evaluations were conducted using 3T MRI scans (Siemens Magnetom 3.0 T XT Numaris/X VA50A‐01NG). The imaging protocol included (Figures 3 and 4):

– *Cartilage thickness measurement:* Performed on proton density‐weighted sequences to assess the grafted area compared to reference cartilage regions (anterior, posterior, contralateral femoral condyle). Sequence parameters: TR 3500 ms, TE 36 ms, slice thickness 3 mm, matrix 464 × 372 pixels in sagittal orientation (Figure 2).
– *T2 mapping:* Quantitative evaluation of cartilage composition and integrity, assessing collagen fibre orientation and water content. Sequence parameters: TR 1910 ms, TE 13.8/27.6/41.4/55.2/69.0 ms, slice thickness 3 mm, matrix 384 × 307 pixels in sagittal orientation (Figure 3).
– *MOCART 2.0 scoring:* Morphological assessment of the repair tissue based on parameters such as defect filling, integration, surface integrity, and subchondral bone status [37].

**Figure 3:**
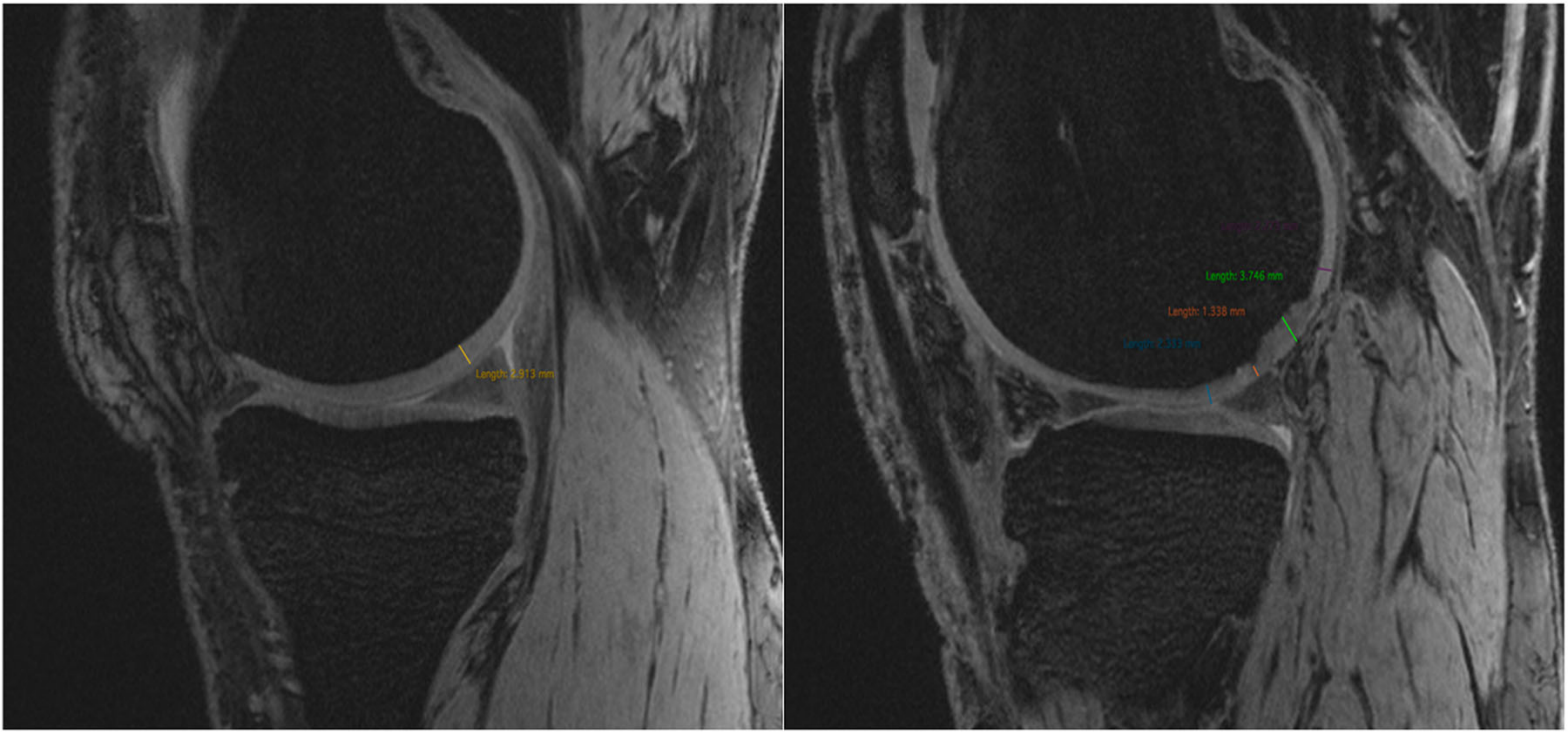
Cartilage thickness measurement. Left: healthy contralateral condyle in the same zone as the lesion on the contralateral defect; right: blue line—thickness directly anterior to the defect; orange line—thickness of the most narrow portion of the defect filling; green line—thickness of the widest portion of the defect filling; purple line‐thickness directly posterior to the defect.

**Figure 4:**
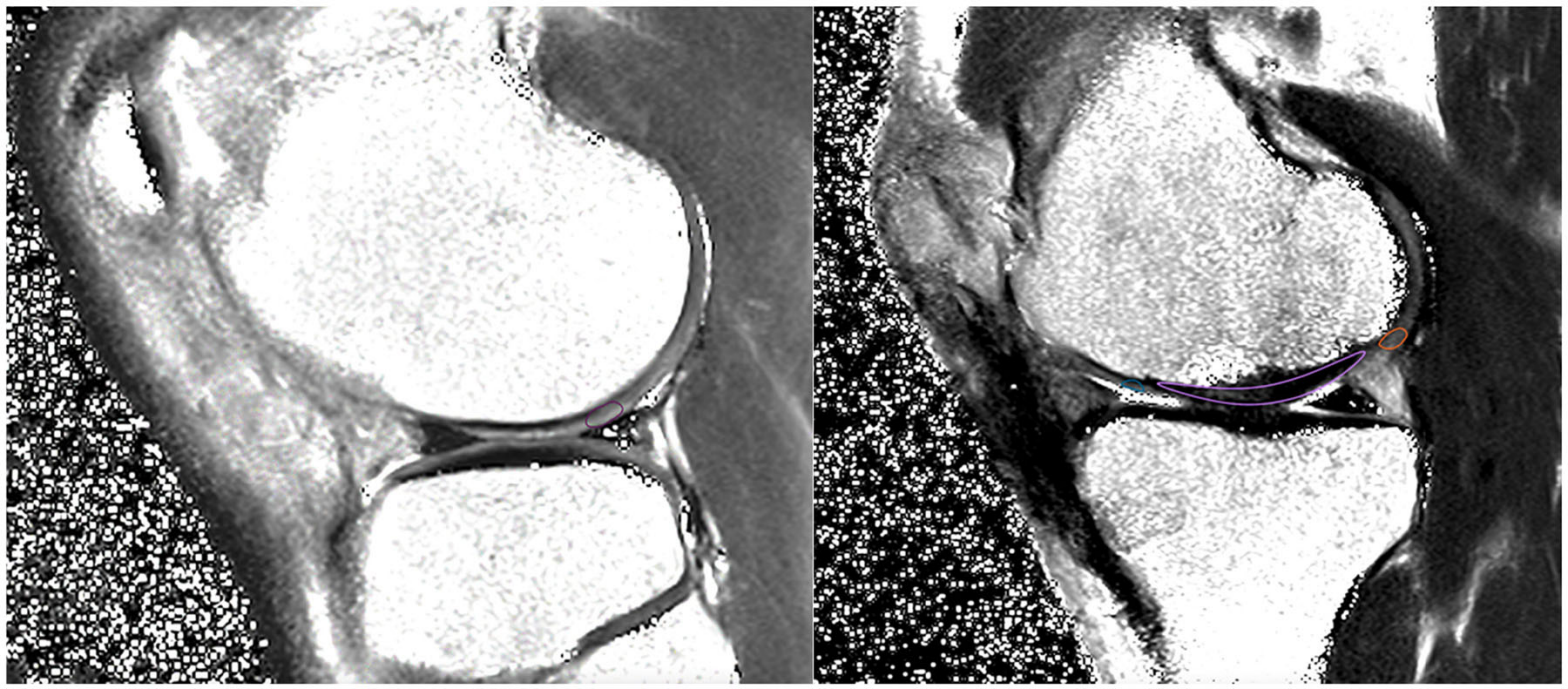
T2 mapping. Left: healthy contralateral condyle in the same zone as the lesion on the contralateral defect; right: blue circle—directly anterior to the defect; purple circle—defect; orange circle ‐ directly posterior to the defect.

All imaging analyses were performed independently by three observers: one experienced musculoskeletal radiologist and two orthopaedic specialists, blinded to clinical outcomes. Interrater reliability was assessed using intraclass correlation coefficients (ICCs).

### Clinical outcome measures

PROMs were collected in person during the clinical visits and at follow‐up appointment, including: International Knee Documentation Committee (IKDC) Score; [22] Knee Injury and Osteoarthritis Outcome Score (KOOS)—all subscales; [36] and Patient‐Reported Outcomes Measurement Information System 29‐Item Profile (PROMIS‐29) [6]—covering physical function, pain interference, fatigue, emotional distress and social participation. Parts of the evaluated PROMs data have been published previously [31].

### Statistical analysis

Statistical analyses were performed using SPSS (Version 29.0; IBM Corp.). Data distribution was assessed with the Shapiro–Wilk test. For normally distributed variables, paired *t*‐tests were applied; nonnormally distributed data were analysed using the Wilcoxon signed‐rank test. Interrater reliability was assessed using the ICC. Correlation analyses between radiological parameters and PROMs were conducted using Spearman's rank correlation coefficient (*r*). Statistical significance was set at *p* < 0.05. An a priori sample size calculation was based on the clinically important difference (CID) for the IKDC after cartilage restoration as previously published (9.2 points) [7]. Assuming a conservative standard deviation (SD) of paired differences of 18, a two‐sided *α* = 0.05 and power = 0.80 yield a required sample size of 25 (*dz* = 0.51).

## RESULTS

The average follow‐up duration between surgery and MRI assessment was 44.4  ±  21.6 months. No adverse events occurred during the procedure, throughout the hospital stay, or during follow‐up.

Radiological assessments revealed promising outcomes following MABCI. The mean cartilage thickness in the treated defect area was 3.4 ± 0.9 mm (range: 1.8–5.00 mm), which was significantly greater than the contralateral femoral condyle (3.0 ± 0.5 mm, range 1.9–3.7 mm, *p* = 0.036) and the anterior reference area (2.6 ± 1.2 mm, range 1.7–3.5 mm, *p* < 0.001), suggesting robust cartilage regeneration. No significant difference was observed when compared to the posterior reference cartilage (2.9 mm ± 0.6 mm, range: 2.1–4.5 mm, n.s.). Interrater reliability for cartilage thickness measurements was excellent (IRR = 0.93), confirming the consistency of these results. T2 mapping demonstrated a mean relaxation time of 50.5 ± 40.0 ms (range 216.0–21.7 ms) in the transplanted cartilage. While no significant differences were observed compared to the contralateral femoral condyle or the anterior reference area, a slight but significant elevation was noted when compared to the posterior reference region (mean 46.11 ms, range: 33.7–58.5 ms, *p* = 0.045). The high interrater reliability (IRR = 0.91) underscored the robustness of these measurements. The MOCART 2.0 scoring system further highlighted satisfactory repair tissue quality, with a mean total score of 61.3 ± 13.0 (range 25.0–78.3). High subscores were observed for volume fill (16.1 ± 3.7/20) and integration with adjacent cartilage (12.9 ± 3.3/15), indicating effective defect coverage and graft stability.

In contrast, lower scores were noted for surface integrity (4.3/10) and tissue structure (1.6/10), suggesting variability in the morphological characteristics of the regenerated cartilage (Figure 5).

**Figure 5:**
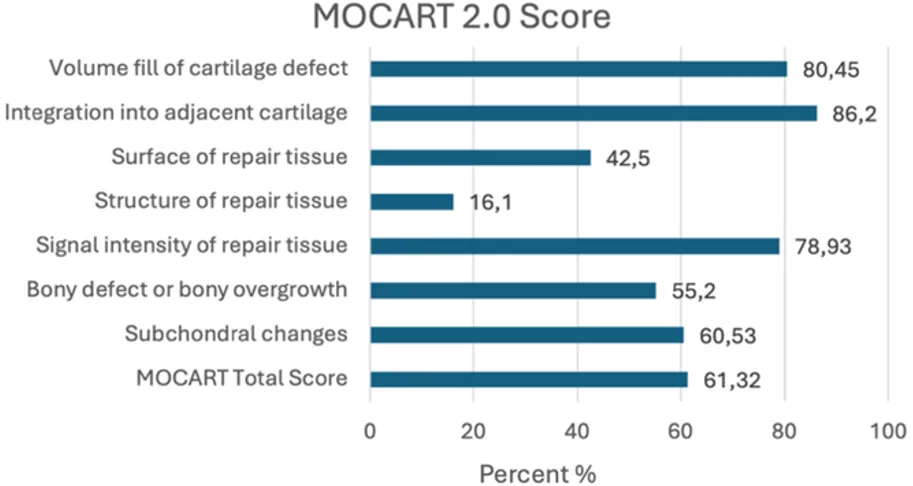
Values for each subscore of the MOCART 2.0. *MOCART 2.0, Magnetic Resonance Observation of Cartilage Repair Tissue.

The IRR for the total MOCART score was excellent (0.871), supporting the reliability of these evaluations.

In terms of the PROMs, there were significant improvements in all scores and their respective subscales from pre‐ to postoperative values (Table 2). The IKDC score improved from a baseline mean of 54.8 ± 17.5 to 70.6 ± 13.2 postoperatively (*p* < 0.001); pain intensity decreased from 5.7 ± 2.3 to 3.5 ± 1.9 (*p* < 0.001), and current knee function improved from 5.4 ± 2.5 to 6.8 ± 1.6 (*p* = 0.004). The KOOS showed significant postoperative improvements across all subscales: ADL increased from 77.6 ± 16.8 to 86.8 ± 14.0 (*p* = 0.003), pain from 66.1 ± 18.6 to 78.7 ± 16.9 (*p* = 0.002), symptoms from 65.8 ± 19.1 to 75.6 ± 16.0 (*p* = 0.006), sport and recreation from 47.3 ± 26.9 to 63.0 ± 24.2 (*p* = 0.009) and quality of life (QoL) from 39.6 ± 17.5 to 51.0 ± 22.7 (*p* = 0.014). The PROMIS‐29 profile revealed significant reductions in pain intensity (5.2 ± 2.4 to 3.0 ± 2.2, *p* < 0.001), as well as improvements in physical function (44.0 ± 6.5 to 48.8 ± 6.3, *p* = 0.002) and social participation (49.4 ± 8.0 to 53.3 ± 6.9, *p* = 0.048). Anxiety, depression and fatigue also showed numerical improvements, though not all reached statistical significance. Patient satisfaction was high, with 92.8% of the cohort satisfied with the procedure, and 81.5% would choose to undergo surgery again.

**Table 2:** Pre‐ to postoperative patient‐reported outcome measure scores comparison.

Score/Subscale	Preoperative mean ± SD	Postoperative mean ± SD	*p*‐value
International Knee Documentation Committee (IKDC) score	54.76 ± 17.51	70.62 ± 13.19	<0.001
IKDC—pain	5.71 ± 2.32	3.54 ± 1.86	<0.001
IKDC—function	5.39 ± 2.45	6.79 ± 1.62	0.004
Knee Injury and Osteoarthritis Outcome Score (KOOS)—ADL	77.61 ± 16.83	86.82 ± 14.03	0.003
KOOS—Pain	66.12 ± 18.59	78.71 ± 16.92	0.002
KOOS—Symptoms	65.79 ± 19.07	75.61 ± 15.97	0.006
KOOS—Sport/Rec	47.32 ± 26.86	63.04 ± 24.17	0.009
KOOS—QoL	39.61 ± 17.47	50.96 ± 22.65	0.014
Patient‐Reported Outcomes Measurement Information System (PROMIS)—Pain	5.22 ± 2.44	3.0 ± 2.16	<0.001
PROMIS—Function	43.96 ± 6.48	48.79 ± 6.26	0.002
PROMIS—Social	49.35 ± 8.01	53.28 ± 6.92	0.048
PROMIS—Anxiety	49.25 ± 9.55	45.87 ± 7.26	n.s.
PROMIS—Depression	48.58 ± 7.45	45.07 ± 6.33	n.s.
PROMIS—Fatigue	45.89 ± 8.12	44.99 ± 8.30	n.s.
PROMIS—Sleep	45.03 ± 7.29	45.48 ± 7.76	n.s.

The correlation analysis between radiological outcomes and PROMs revealed several significant associations. Higher MOCART scores correlated with improved mental health domains in the PROMIS‐29, including lower postoperative anxiety (*r* = 0.595, *p* < 0.001) and depression (*r* = 0.460, *p* = 0.005), suggesting that better morphological repair is associated with psychological well‐being (Table 3).

**Table 3:** Correlation analysis between radiological outcomes and patient‐reported outcome measure scores.

Parameter pair	Correlation coefficient (*r*)	*p*‐value
Cartilage thickness and International Knee Documentation Committee (IKDC) pain	−0.457	0.013
Cartilage thickness and Knee Injury and Osteoarthritis Outcome Score (KOOS) quality of life	0.429	0.020
Cartilage thickness and Patient‐Reported Outcomes Measurement Information System (PROMIS) pain intensity	−0.401	0.031
Cartilage thickness and PROMIS depression	−0.449	0.015
Thickness and PROMIS fatigue	−0.425	0.022
Thickness and PROMIS social roles	0.481	0.008
Magnetic Resonance Observation of Cartilage Repair Tissue (MOCART) and PROMIS anxiety	0.595	<0.001
MOCART and PROMIS depres	0.46	0.005
MOCART and PROMIS social	−0.368	0.049
T2 mapping and PROMs	n.s.	n.s.

Additionally, greater cartilage thickness was significantly associated with reduced pain intensity (*r* = –0.457, *p* = 0.013) and improved QoL (*r* = 0.429, *p* = 0.02), highlighting the clinical relevance of structural repair integrity.

## DISCUSSION

The most important findings of this study indicate that the MABCI procedure for the treatment of osteochondral femoral defects yields satisfactory radiological results with significant improvements in cartilage thickness, graft integration and physiological T2‐mapping values. Furthermore, significant improvements were seen in all PROMs and their subscales analysed, indicating a high level of patient satisfaction and subjective QoL. Importantly, there were significant correlations between specific radiological parameters and PROMs, reflecting the clinical relevance of structural repair characteristics.

The observed mean cartilage thickness of 3.4 mm in the treated defects significantly exceeded that of the contralateral reference cartilage, suggesting robust hyaline‐like tissue regeneration. In this context, it should be acknowledged that increased cartilage thickness may also indicate pathologic hypertrophic changes. However, the relatively small differences observed in the analysed cohort make a clinically relevant hypertrophy less likely. This finding is consistent with the results of a recent literature review [30], who reported favourable outcomes in ABCI‐treated defects in 18 studies involving 344 patients with a mean follow‐up of 73.2 months. In addition, the positive correlation between cartilage thickness and PROMs ‐ specifically reduced pain intensity and improved QoL‐reinforces the functional relevance of structural integrity. Similar associations were reported by Weishorn et al. [40], where cartilage morphology as assessed by MOCART correlated moderately with KOOS scores, particularly in domains related to activities of daily living and sports function. T2 mapping in the examined cohort showed increased relaxation times in the grafted cartilage compared to the posterior reference area, reflecting biochemical differences that may indicate ongoing remodelling [33].

The mean MOCART 2.0 score of 61.3 is consistent with previously reported results following advanced cartilage repair techniques [15, 16, 35]. High subscores for defect filling and integration with adjacent cartilage indicate effective structural repair, while lower scores for surface integrity and tissue structure reflect persistent heterogeneity in the repair tissue, a known phenomenon following biological cartilage repair techniques [15, 21, 29, 35].

However, the subchondral bone subscore within the MOCART 2.0 system was notably among the lowest‐rated categories in the analysed cohort, as visualised in Figure 2, reflecting persistent irregularities despite otherwise favourable graft integration. This observation aligns with findings from Ebert et al. [12, 13, 15], who demonstrated that the severity of preoperative subchondral bone marrow oedema showed no significant association with postoperative patient‐reported pain, symptoms, or MRI‐based graft repair parameters following MACI. These results support the hypothesis that certain subchondral alterations—while radiologically detectable—may not directly influence clinical recovery or functional outcomes. Consequently, the low subchondral bone subscores observed in the analysed cohort may reflect structural remodelling or bone signal changes of uncertain clinical impact, rather than failure of the osteochondral repair unit. In contrast, Jung et al. [23] recently demonstrated that specific subchondral MRI biomarkers, including bone marrow lesion volume and water fraction, were independently associated with pain outcomes at 1 year after MABCI, suggesting that distinct subchondral tissue characteristics may variably influence symptom persistence depending on lesion phenotype and timing. This highlights the nuanced and multifactorial nature of subchondral involvement in osteochondral repair and underscores the need for further stratified analyses based on imaging biomarkers.

Interestingly, the analysis showed significant correlations between MOCART scores and mental health domains within PROMIS‐29, including postoperative anxiety and depression. Retzky et al. [35] also identified MOCART as a predictor of achieving a patient acceptable symptomatic state (PASS), particularly when scores exceeded 55 points. These findings suggest that morphological graft characteristics may reflect functional benefits and influence subjective well‐being on an individual and patient‐relevant plane. However, their predictive value for functional outcomes remains variable and not fully understood.

The reported significant improvements in PROMs are consistent with results reported after advanced cartilage repair techniques. In particular, the mean postoperative IKDC score of 70.62 is in line with the findings of Ebert et al. [12, 13], where IKDC improvements after MACI procedure ranged from 65 to 75 over comparable follow‐up periods. Similarly, the observed gains in the KOOS subscales, particularly pain and sport/recreation, are consistent with the findings of Hoburg et al. [19, 20], who reported mean KOOS improvements of 20–25 points following spheroid‐based MACI. The KOOS score outcomes in the present cohort were almost identical to those reported by Weißhorn et al. in their registry study, in which MABCI demonstrated slightly superior subjective outcomes compared to ACI alone [39], Notably, the increase in the KOOS—QoL subscale from 39.61 to 50.96 was less pronounced compared to the data from another study from Weishorn et al., where QoL scores exceeded 60 points at long‐term follow‐up [40]. This difference may reflect differences in defect characteristics, since this study focused specifically on chondral defects without subchondral bone integrity loss.

High patient satisfaction data further support the clinical efficacy of MABCI, with over 80% of patients stating that they would choose the procedure again. These results are comparable to the generally high satisfaction rates reported for other advanced cartilage repair techniques, including MACI and OATS [5, 8, 9, 10, 11, 12, 13, 32]. Despite statistically significant improvements, mean PROM values such as an IKDC of 70.5 and VAS pain of 3.0 at follow‐up still indicate residual functional limitations. Similar findings have been reported for other techniques including OAT, and OCA, with some studies showing slightly superior results and others reporting somewhat less favourable outcomes [2]. Although failure rates were low, the persistence of symptoms in a subset of patients highlights the multifactorial nature of knee function, where factors beyond cartilage integrity, such as proprioception, muscle strength and psychosocial elements, play a critical role.

This study has several strengths, including the comprehensive radiological assessment utilising advanced MRI techniques and the integration of various PROMs covering most aspect of QoL and subjective functional assessment. However, certain limitations must be acknowledged. The retrospective design and modest sample size may limit the generalisability of the findings of the present study. While the MOCART score provides valuable structural insights, it may not fully capture the biochemical or biomechanical properties of the repair tissue. Moreover, the analysed mid‐term follow‐up may not fully capture the long‐term outcome. Additionally, the lack of baseline imaging must be considered. Importantly, in this study it was able to identify correlations between radiological parameters and PROMs, but these need to be confirmed in future studies with larger cohorts [28]. Finally, the absence of a control group precludes direct comparisons with alternative treatment modalities.

Taken together, MABCI demonstrated favourable radiological and clinical outcomes in the treatment of osteochondral defects of the knee with a high cartilage thickness, good integration and physiological T2‐mapping values. The correlation between radiological parameters and PROMs suggests that tissue quality relates closely to patient outcome. This underlines the value of radiological assessment as a surrogate for clinical benefit. Clinically, these findings support MABCI as a relevant option for young, active patients with osteochondral defects. Future studies with larger cohorts and longer follow‐up periods are warranted to validate these findings and to explore the mechanistic underpinnings of the observed associations.

## CONCLUSION

MABCI demonstrates radiologically confirmed cartilage and subchondral bone regeneration in the treatment of osteochondral defects of the femoral condyle, accompanied by substantial improvements in patient satisfaction and quality of life.

## AUTHOR CONTRIBUTIONS

All authors contributed to the study conception and design. Material preparation, data collection and analysis were performed by Stephan Oehme, Janne Callsen and Danko D. Milinkovic. The first draft of the manuscript was written by Stephan Oehme and Danko D. Milinkovic and all authors commented on previous versions of the manuscript. All authors read and approved the final manuscript.

## CONFLICT OF INTEREST STATEMENT

Danko D. Milinkovic is currently a member of the KSSTA editorial board and otherwise has no conflicts of interest to disclose. No benefits in any form have been or will be received from any commercial party directly or indirectly related to the subject matter of this article. The remaining authors declare no conflict of interest.

## ETHICS STATEMENT

Ethical approval was obtained from the local ethics committee (Ethikkommission der Charité –Universitätsmedizin Berlin; Nr: EA4/220/20), and all participants provided written informed consent. The study was conducted in accordance with the Helsinki Declaration.

## Data Availability

Raw data can be made available upon request.
